# Association of Selected Cardiovascular Markers With Tuberculosis: Community-Based Exploratory Cross-Sectional Analytical Study in Puducherry

**DOI:** 10.7759/cureus.42343

**Published:** 2023-07-23

**Authors:** Premkumar Ramasubramani, Sitanshu Sekhar Kar, Sonali Sarkar, Vir Singh Negi, Santhosh Satheeh, Madhusmita Mohanty Mohapatra, Kavadichanda Chengappa

**Affiliations:** 1 Department of Preventive and Social Medicine, Jawaharlal Institute of Postgraduate Medical Education & Research, Puducherry, IND; 2 Department of Clinical Immunology, Jawaharlal Institute of Postgraduate Medical Education & Research, Puducherry, IND; 3 Department of Cardiology, Jawaharlal Institute of Postgraduate Medical Education & Research, Pondicherry, IND; 4 Department of Pulmonary Medicine, Jawaharlal Institute of Postgraduate Medical Education & Research, Puducherry, IND; 5 Department of Clinical Immunology, Jawaharlal Institute of Postgraduate Medical Education & Research, Pondicherry, IND

**Keywords:** radiology & imaging, inflammatory cytokines, interleukins, cardio vascular disease risk reduction, cardio vascular disease, tuberculosis(tb)

## Abstract

Introduction

India accounts for one-fourth of the global tuberculosis (TB) burden and also faces a rising burden of non-communicable diseases. Only a few have studied the association between the infective pathogenesis of TB and cardiovascular diseases (CVD).

Methods

A cross-sectional exploratory analytical design was used to compare CVD risk factors and immunological and radiological parameters. This was a pilot study conducted in two primary health centers in urban Puducherry between February 2020 and March 2021. Household contacts (HHC) were either spouses or siblings of the newly diagnosed pulmonary tuberculosis (PTB) patients selected for comparison as their exposure to infection would be similar to those who were diseased yet did not develop illness. Assuming a difference of 5% in CVD risk between the general population and TB patients, with a 95% confidence interval, the sample size calculated was 153 in each group by nMaster v2.0. Considering the feasibility and resource constrain, we recruited 50 newly diagnosed PTB patients, their age- and gender-matched 50 HHC and 50 PTB patients who completed treatment a year before. CVD risk factors were compared using chi-square or Fisher exact test. Interleukins-6 (IL-6), interferon-gamma (INF-γ), highly specific - C reactive protein (hs-CRP), and carotid intima-media thickness (CIMT) were compared using ANOVA or Kruskal-Wallis test.

Results

Most participants from each group belonged to lower socio-economic strata and were males (40/50). Alcohol intake was higher among newly diagnosed and treatment-completed PTB patients (82.5% vs 72.5%). Excess salt intake (58%) was present more in newly diagnosed PTB patients. General and abdominal obesity were seen more among HHC (64% and 84%) and treatment-completed PTB patients (50% and 74%). IL-6 was higher in newly diagnosed PTB patients, whereas INF-γ and hs-CRP were higher in treatment-completed PTB patients. The largest proportion of those having high CIMT values was also in the treatment-completed PTB patients.

Conclusion

Levels of immune markers hint at the role of inflammation due to TB disease being related to the high CIMT values among the newly diagnosed and treatment-completed PTB patients. CVD risk was higher among TB patients even if they had completed treatment and were declared cured.

## Introduction

Tuberculosis (TB) contributed to 9.9 million incident cases which led to over 1.3 million deaths in the year 2020 [1]. Known as the TB hub of the world, India accounted for 26% of the global TB burden. Though tremendous efforts were taken to control the spread, the estimated incident cases of TB in India were 2.6 million in the year 2020 [2].

Another major cause of mortality was non-communicable diseases (NCD), which showed a rising trend with over 40 million deaths per year, of which 15 million deaths are among individuals less than 70 years of age [3]. NCDs severely affect low- and middle-income countries like India, where it contributed to 78% (31 million) of all deaths [4].

The immunity reaction contains the manifestation of TB disease by granuloma formation in the lungs. The mediators of immune response produce inflammatory markers like tumor necrosis factors, interleukins, and interferons [5]. Clinical evidence and experimental models have shown amplified immune response in TB-infected individuals with higher levels of several inflammatory cytokines disrupting homeostasis [6].

India is facing a dual epidemic of TB and CVD. Studies have been conducted to see if the infective pathology of TB paves the way for the onset of CVDs. A nationwide study from Taiwan showed the risk of subsequent acute coronary syndrome (ACS) in patients with newly diagnosed pulmonary tuberculosis patients (PTB) was 40% higher than the non-infected individuals [7]. TB patients had twice the higher risk of getting an ischemic stroke than non-TB patients [8].

Elevated levels of cytokines like interleukin-6 (IL-6) are related to the underlying inflammatory process [9]. Interferon-gamma (INF-γ) can differentiate TB infection from TB disease, with higher levels being documented in the diseased state, which decreases with the treatment [10]. C-reactive proteins (CRP), produced in response to IL-6, are another marker in serum found to be high in those with PTB [11]. These inflammatory markers are also related to atherogenesis and plaque formation. Pathogenesis of CVD is mediated through the condition of blood vessels in the body. Vascular conditions, which can be easily assessed can serve as an indicator of cardiac status [12]. Therefore, in addition to the traditional risk factors like age, gender, family history, history of NCDs, obesity, tobacco consumption, physical inactivity, and dyslipidemia, which have been extensively studied for various populations around the globe, we proposed carotid intima-media thickness (CIMT) changes in TB patients as a marker of CVD risk. One way of identifying the changes that happen due to the disease is by comparing these markers among those without the disease but having similar socioeconomic and environmental risk factors such as household contacts (HHC). This knowledge is necessary to explore ways of controlling inflammation as a possible reduction of the risk of CVD. Therefore, in this exploratory study, we compared the risk factors of CVD, inflammatory cytokines such as IL-6 and INF-γ, high sensitive C-reactive proteins (hs-CRP), and CIMT between the newly diagnosed PTB patients, their HHC, and PTB patients who have completed treatment for more than a year.

## Materials and methods

An exploratory cross-sectional analytical study was conducted between March 2020 and February 2021 in two high TB burden primary health centers (PHC), in the Union Territory of Puducherry, India. Under National Tuberculosis Elimination Programme, services were delivered through the designated microscopy centers (DMCs) and the peripheral health institutions with TB units (TUs) at the subdistrict level. Under the program, free-of-cost diagnosis, as well as treatment, was provided. TB diagnosis would be done at DMCs and get referred to PHC in their locality for treatment. The sociodemographic details, clinical presentation, and investigation findings were maintained at the PHC as a TB register and followed up using TB treatment cards.

Study participants were included in three groups - group 1: newly diagnosed adult PTB patients, group 2: HHC of the newly diagnosed adult PTB patients, and group 3: PTB patients who had completed treatment one year before the commencement of the study. Participants in group 1 were recruited within four weeks of initiation of treatment, to control the effect of anti-TB drugs on study findings. Participants with a self-reported history of hypothyroidism and other immunological diseases like systemic lupus erythematosus, rheumatoid arthritis, or osteoarthritis were excluded to minimize the interference with the immunological parameters. If either newly diagnosed adult PTB patients or their respective HHC did not express willingness, the other was not recruited to maintain the link between groups 1 and 2.

The study was approved by the Institute Ethics Committee and State TB - Operational Research Committee under the program. Written consent was obtained before enrolling the study participants and consecutive sampling was incorporated. Data were collected regarding the socio-demographic variables like age, gender, education, occupation, and marital status and using the WHO STEPS core questionnaire tobacco use, alcohol consumption, diet, physical activity, history of raised blood pressure, diabetes, raised total cholesterol, and anthropometry at the PHC were noted [13]. The blood sample was collected within a week of recruitment and serum was stored at -80°C for estimation of IL-6, INF-γ, and hs-CRP. The samples were only thawed once immediately before the bio-analysis. In the same visit, the measurement of CIMT was measured by the radiologist using an ultrasound transducer.

Assuming a difference of 5% in CVD risk between the general population and TB patients, with a 95% confidence interval, the sample size calculated was 153 in each group by nMaster v2.0. Considering the feasibility and resource constrain, 50 newly diagnosed PTB patients were recruited into group 1, and 100 healthy age- and gender-matched individuals (50 HHC and 50 previously treated PTB patients) were recruited into groups 2 and 3.

Data was collected in EpiCollect5 and analyzed using STATA v14.2. Age, lipid profile immunological markers, and radiological markers were summarized as mean (SD) or median (IQR) based on distribution. Biochemical parameters like glycated hemoglobin (HbA1C) levels, lipid profile (total cholesterol, triglycerides, low- and high-density lipoprotein), immunological parameters, and CIMT measures, being continuous variables were compared between the groups either using ANOVA or Kruskal-Wallis test as appropriate. Gender, marital status, family status, socio-economic status as per the Modified Kuppusamy scale, religion, diet, family history of diabetes mellitus and hypertension, tobacco use, and alcohol use were summarized as frequencies (proportions). WHO/ISH risk charts were used to predict 10-year fatal or non-fatal CVD risk summarized as percentages [14,15]. Chi-squared test and Fisher exact test were used to compare these parameters between groups.

## Results

We contacted 67 newly diagnosed PTB patients and their HHC, and 96 treatment-completed PTB patients to enroll 50 participants in each group. The response rate was 74.6% among the newly diagnosed PTB patients and their HHC, and 52.1% among treatment-completed PTB patients.

The mean (SD) age (years) of the newly diagnosed PTB patients, their HHC, and treatment-completed PTB patients were 38.4±6.8, 36.6±5.3, and 38.9±7.1, respectively. Almost three-fourths of the participants were less than 45 years of age. The majority of the study participants were males (80%) and were literate. More than 35% of the participants had a graduation/post-graduation degree. The majority belonged to the lower-middle and upper-middle socio-economic class as measured by the modified Kuppusamy scale. Over 60% of the participants belonged to nuclear families and more than three-fourths of them were married (Table 1).

**Table 1:** Socio-demographic characteristics of the study participants, N=150 Group I: newly diagnosed tuberculosis patients; Group II: household contacts of newly diagnosed tuberculosis patients; Group III: treatment-completed tuberculosis patients *Modified Kuppusamy scale

Characteristics	Group I (n=50) n (%)	Group II (n=50) n (%)	Group III (n=50) n (%)
Age categories (years)			
<35 years	20 (40)	12 (24)	15 (30)
>35 years	30 (60)	38 (76)	35 (70)
Gender			
Male	40 (80)	40 (80)	40 (80)
Female	10 (20)	10 (20)	10 (20)
Educational status			
No formal schooling	4 (8)	2 (4)	6 (12)
Primary (1-4)	6 (12)	7 (14)	9 (18)
Secondary (5-10)	12 (24)	13 (26)	12 (24)
Higher secondary (11-12)	6 (12)	11 (22)	6 (12)
Graduate /post-graduate	22 (44)	17 (34)	17 (34)
Socioeconomic status*			
Upper middle (II) (16-25)	9 (18)	9 (18)	6 (12)
Lower middle (III) (11-15)	26 (52)	26 (52)	36 (72)
Upper lower (IV) (5-10)	14 (28)	14 (28)	7 (14)
Lower (V) (<5)	1 (2)	1 (2)	1 (2)
Type of family			
Nuclear	32 (64)	32 (64)	28 (56)
Joint	13 (26)	13 (26)	15 (30)
Three generations	5 (10)	5 (10)	7 (14)
Marital status			
Unmarried	9 (18)	7 (14)	6 (12)
Married	40 (80)	43 (86)	43 (86)
Widow/ widower/divorced/separated	1 (2)	0 (0)	1 (2)
Details of co-morbidities			
Diabetes	25 (50)	21 (42)	18 (36)
Hypertension	19 (38)	22 (44)	23 (46)
Dyslipidemia	19 (38)	21 (42)	24 (48)

There were no current smokers among newly diagnosed PTB patients, more than one-fourth of their HHC were current smokers. There were no tobacco users among women. Current alcohol use was higher among PTB patients (82.5%) and treatment-completed TB patients (72.5%). Nearly three fourth of the newly diagnosed PTB patients were consuming inadequate amounts of fruits and vegetables, which was much higher among the HHC (90%) and treatment-completed TB patients (80%). The practice of adding salt right before eating and eating processed food was higher among newly diagnosed PTB patients. A higher proportion of newly diagnosed PTB patients (72%) had low physical activity compared to the HHC (46%) and treatment-completed PTB patients (50%). Two PTB patients were underweight. Overweight and obese were more among the HHC (64%) than the treated group (50%) and PTB group (42%). Abdominal obesity was also highest in the HHC (84%) followed by treatment-completed patients (74%). Raised blood pressure was higher among the HHC (18%) compared to the newly diagnosed PTB patients (12%) and treatment-completed group (14%). Raised HbA1C (≥6.5%) was seen in six newly diagnosed PTB patients, ten HHC, and nine treatment-completed PTB patients (Table 2).

**Table 2:** Behavioural risk factors among study participants, N=150 *Chi-square test ^#^Fisher exact test ^+^Consumption of fewer than five servings of fruits and vegetables per day ^$^Low physical activity: <600 METS-minutes, active: 600 to 1800 METS-minutes, highly active: >1800 METS-minutes ^@^WHO Asian Pacific guidelines for the classification of obesity ^†^International diabetes federation criteria for country-specific waist circumference values for South Asia (waist circumference of ≥80cm in males and ≥90cm in females are considered to have central obesity)

Risk factors	Group I (n=50) n (%)	Group II (n=50) n (%)	Group III (n=50) n (%)	P-value
Tobacco use (male gender alone, N=120)				
Current tobacco use (past one month)	0 (0)	13 (32.5)	9 (22.5)	0.01^#^
Ever used tobacco	6 (15)	17 (42.5)	16 (40)	0.02*
Alcohol use (male gender alone, N=120)				
Current alcohol use (past one year)	33 (82.5)	23 (57.5)	28 (72.5)	0.44*
Ever used alcohol	27 (67.5)	33 (82.5)	31 (77.5)	0.80*
Fruits and vegetable intake				
Inadequate intake of fruits and vegetable^+^	37 (74)	45 (90)	40 (80)	0.12*
Dietary salt intake				
The practice of eating processed food/adding salt right before eating	29 (58)	19 (38)	20 (40)	0.08*
Physical activity^$^				
Low physical activity	36 (72)	23 (46)	25 (50)	0.02*
Active/highly active	14 (28)	27 (54)	25 (50)	
Body mass index^_@_^ categories				
Underweight (<18.5 kg/m^2^)	2 (4)	0 (0)	0 (0)	0.14^#^
Normal (18.5-22.9kg/m^2^)	27 (54)	18 (36)	25 (50)	
Overweight (23-24.9 kg/m^2^)	20 (40)	25 (50)	21 (42)	
Obese (>25 kg/m^2^)	1 (2)	7 (14)	4 (8)	
Abdominal obesity^†^				
Present	25 (50)	42 (84)	37 (74)	0.01^#^
Absent	25 (50)	8 (16)	13 (26)	
Hypertension status				
Raised blood pressure	6 (12)	9 (18)	7 (14)	0.77*
Diabetes status				
Raised HbA1C (≥6.5)	6 (12)	10 (20)	9 (18)	0.62*

Total cholesterol levels (mg/dl) were higher among the newly diagnosed PTB patients (219.84±46.40) compared to that of HHC (208.15±46.68) and treatment-completed PTB patients (209.55±68.76). LDL values (mg/dl) were higher in both newly diagnosed PTB patients (127.82±33.40) and treatment-completed PTB patients (126.57±47.53) compared to HHC (120.85±40.63). Triglyceride levels (mg/dl) were higher among treatment-completed PTB patients. Median IL-6 values (pg/ml) were also higher in PTB patients than that in HHC and the treatment-completed groups (35.8 vs 22.0 vs 23.1). Median INF-γ levels (pg/ml) were more or less equal among the newly diagnosed PTB patients and their HHC. But treatment-completed PTB patients had a higher level (56.8 vs 56.7 vs 72.6). Hs-CRP values (mg/l) also showed a similar trend as INF-γ among the three groups (3.5 vs 3.0 vs 5.7). CIMT measure was slightly higher in the PTB patients and the already treated groups as compared to HHC, the mean+SD values (mm) being 0.6±0.12, 0.64±0.17. and 0.65±0.19 in the HHC, PTB patients, and the treatment-completed groups, respectively (Table 3).

**Table 3:** Lipid levels and immunological and radiological parameters of study participants, N=150 Group I, newly diagnosed tuberculosis patients; Group II, household contacts of newly diagnosed tuberculosis patients; Group III, treatment-completed tuberculosis patients *ANOVA test; ^#^Kruskal-Wallis test HbA1C, glycated hemoglobin; TC, total cholesterol; LDL, low density lipoprotein; HDL, high density lipoprotein; TGA, trigliceride; IL-6, interleuikin-6; INF-γ, interferon gamma; hs-CRP, high sensitive C-reactive protein; CIMT, carotid intimal medial thickness

	Group I (n=50) n (%)	Group II (n=50) n (%)	Group III (n=50) n (%)	P-value
HbA1C (%)	6.05±1.5	6.0±1.4	5.9 ± 1.3	0.84*
TC (mg/dl)	219.8±46.4	208.2±46.7	209.6 ± 68.8	0.51*
LDL (mg/dl)	127.8±33.4	120.9±40.6	126.6 ± 47.5	0.66*
HDL (mg/dl)	42.4±6.3	37.6±8.3	40.5±6.5	0.01*
TGA (mg/dl)	176.5 (151.0-247.0)	181.7 (95-230.2)	231.8 (87.0-272.5)	0.35^#^
IL-6 (pg/ml)	35.8 (19.1-57.9)	22.0 (7.3-59.6)	23.1 (10.2-34.2)	0.01^#^
INF-γ (pg/ml)	56.8 (49.5-80.2)	56.7 (27.1-63.6)	72.6 (50.5-92.3)	0.04^#^
hs-CRP (mg/l)	3.5 (1.6-6.6)	3.0 (1.6-4.3)	5.7 (1.9-10.2)	0.02*
CIMT	0.6±0.2	0.6±0.1	0.6±0.2	0.45*

**Figure 1:**
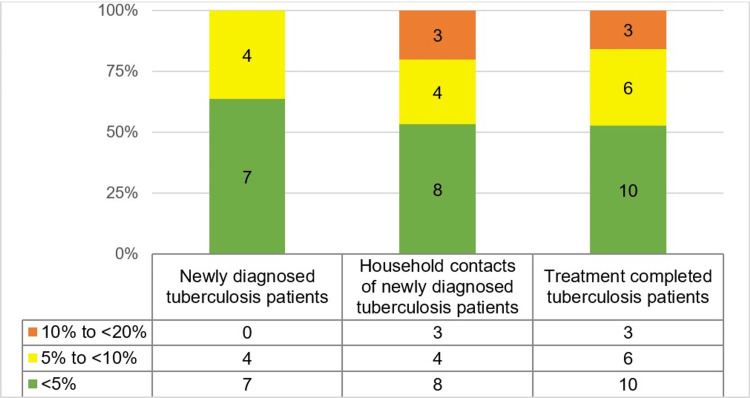
Distribution of 10-year risk of fatal and non-fatal cardiovascular events using WHO cardiovascular disease risk laboratory-based charts WHO/ISH risk charts were used to predict 10-year fatal or non-fatal CVD risk WHO, World Health Organization; ISH, International Society of Hypertension; CVD, cardiovascular diseases

Assessment of ten-year risk of fatal and non-fatal CVD risk based on WHO CVD risk laboratory-based charts was carried out for participants who were more than 40 years of age. CVD risk was higher among the treatment-completed PTB patients; 10-<20% risk was predicted in three participants, 5-<10% risk for six, and <5% for ten. A fairly similar proportion of newly diagnosed PTB patients and their HHC had <5% risk (63% and 53%). CVD risk of 10 to <20% was present in three of the HHC and treatment-completed PTB patients, which was higher than the newly diagnosed PTB patients (Figure 1).

## Discussion

In behavioral risk factors, we found that a higher proportion HHCs had tobacco use than PTB patients in our study. This could be because of the change in behavior of the TB patients due to their disease status. Current alcohol use was higher in the PTB patients and treatment-completed TB patients as the prevalence is high in the Union Territory of Puducherry, which was an incidental finding [16]. Low physical activity was noted among 72% of the newly diagnosed PTB patients, in comparison, only half of their HHCs and treatment-completed PTB patients had low physical activity. This might be due to the TB disease-causing lethargy and weakness, which were one of the presenting symptoms of the patients [17]. Inadequate physical activity is a risk factor for CVD as well [18]. Inadequacy in nutrition is associated with an increased risk of acquiring infection and activation of TB disease [19,20]. In our study, we found that inadequate consumption of fruits and vegetables was more among the HHCs (90%) and treatment-completed PTB patients (80%) than the newly diagnosed PTB patients. This difference might be due to the effect of the Nikshay Poshan Yojana or Direct Benefit Transfer scheme where cash incentive was provided for nutritional support for TB patients and also the effect of the health worker's intervention in terms of nutritional advice [21].

Raised HbA1C levels (≥6.5%) were seen among 20% HHCs, which was more than the newly diagnosed PTB patients (12%). Ginandjar et al. showed the prevalence of uncontrolled glucose was 29% among TB patients in Indonesia [22]. However, in India, only 5% had deranged glucose values as per the TB-Diabetes Report 2017 [23]. One reason for more participants having deranged blood glucose than the national average could be due to the use of HbA1C levels to assess control status whereas in the program the serum blood sugar levels were assessed. About 18% of the treatment-completed PTB patients had raised HbA1C levels. However, the differences (12% vs 20% vs 18%) were not statistically significant.

Total cholesterol levels were higher than the normal cut-off of 200 mg/dl in all three groups. LDL levels were also higher than 100 mg/dl in all three groups. Newly diagnosed PTB patients had a higher mean TC and LDL than their HHCs as well as the treatment-completed PTB patients. We see the proportional relationship between the degree of disease and the TC levels similar to the Volpato et al. study [24]. In the case of HDL cholesterol, studies showed that TB patients had higher levels of HDL [25]. In our study, unexplained lower levels of HDL were seen in the newly diagnosed PTB patients followed by treatment-completed PTB patients compared to HHCs.

The mean IL-6 values were higher among the newly diagnosed PTB patients compared to their HHCs (35.84 pg/ml vs 22.01 pg/ml). Elevated IL-6 is related to the underlying inflammatory process, which is a promising marker as mentioned by Anbarasu et al. [9]. And in TB diseases, elevated IL-6 induces early INF-γ production [26]. In our study, we found elevated levels of IL-6 as found in hypertensive patients (the study by Miguel et al.) [6]. INF-γ can differentiate TB infection from TB disease, the reliability being very high and thus mentioned as the protective factor [10]. In our study, INF-γ was similar in newly diagnosed PTB patients and their HHCs but the levels were higher among treatment-completed PTB patients. This shows the clear link between IL-6 and INF-γ. Also, the presence of INF-γ is the marker of a disease process that can lead to enhanced oxidative stress causing CVD [27]. Amelio et al. observed that the level of CRP in serum was high in those with PTB when compared to those without the disease [11]. In our study, we had comparable results. The mean value of the hs-CRP among newly diagnosed PTB patients was 3.54 mg/L, which was higher than their HHCs (3.06 mg/L). It was notable that treatment-completed PTB patients had much higher levels of hs-CRP. We observed a gradual rise in the hs-CRP from non-exposed to diseased and treatment being completed. To our knowledge, such a trend has not been documented earlier. This shows that the inflammatory process continues even if patients have been declared cured after the completion of the treatment. Not to forget that these immunological markers were related to various other diseases from communicable diseases or NCD or autoimmune disorders as well.

The CIMT values were below the cut-off of 0.9 mm to label them as thickened carotid walls or the presence of atherosclerotic plaque but were closer to the mean values found in the case of recurrent common infections, 0.67 mm to 0.69 mm as reported by Priscilla et al. [28]. There was a gradual rise in the values as we move from HHCs to newly diagnosed PTB patients to treatment-completed PTB patients from 0.61 mm to 0.64 mm to 0.65 mm. The vascular changes were the penultimate stage of CVD [29]. And this clearly showed that the pathological changes related to CVD were higher among the PTB patients and also treatment-completed PTB patients. The worrying trend was the higher levels of LDL and lower levels of HDL, along with very high levels of hs-CRP and the presence of behavioral factors like tobacco and alcohol use with lower fruits and vegetables intake may lead to a higher risk of cardiovascular events among the treatment-completed PTB patients, who often do not get the medical attention as the newly diagnosed PTB patients.

Corroborating evidence showed more than 10% CVD risk, which was higher among the treatment-completed PTB patients (47%) suggesting that the inflammatory changes starting with TB disease continue even after completion of treatment and cure rendering them susceptible to fatal or non-fatal CVD events. Among the PTB patients, it was 36%. Among HHCs, it was 46%, which was much higher than the 17% risk estimated by Ghorpade et al. in the general population [30]. This may be because more were overweight and obese among HHC.

Strengths and limitations

This is one of the few studies to find the association of CVD risk in TB patients, which has assessed behavioral and biochemical parameters along with the immunological and radiological parameters. To our knowledge, this is the first of its kind in the South Indian population. TB patients were compared with their HHC since similar socioeconomic and environmental risk factors would be common in them and the comparison would be robust. 

Social desirability bias was noted while documenting the tobacco use among the participants, i.e., TB patients would have not revealed their true smoking status. Since this was a cross-sectional study, we were not able to assess the temporal relation. Due to the small sample size, we were not able to get significant statistical significance in the comparisons of the immunological and radiological parameters and also, we encountered incidental findings like a higher proportion of participants with a CVD risk of more than 10%. The non-specific nature of the immune mediators validates a panel of immunological markers to be studied. And the ongoing pandemic due to SARS-CoV-2 during this study would have immunologically medicated disease pathogenesis and response that might affect the blood parameters.

## Conclusions

Higher levels of cytokines in PTB hint at the role of inflammation due to TB disease. This prompts a panel of immunological markers to be studied for a clear link. Relatively higher CIMT is proposed to be due to inflammation in both new and treatment-completed PTB patients, which causes atherosclerotic plaque, the major reason for all CVD. This study's results also showed that the CVD risk was higher among the TB patients even if they had completed treatment and declared cured, which needs further exploration. Therefore, it is not the disease to be treated but also the underlying disrupted homeostasis. A robust study with a larger sample size and a long-term follow-up period needs to be carried out to establish the link between TB disease pathogenesis and CVD risk. This will broaden our limited knowledge and expand the screening program for co-existing diseases with TB.
